# Characterization of the Complete Mitochondrial Genome of *Leucoma salicis* (Lepidoptera: Lymantriidae) and Comparison with Other Lepidopteran Insects

**DOI:** 10.1038/srep39153

**Published:** 2016-12-15

**Authors:** Yu-Xuan Sun, Lei Wang, Guo-Qing Wei, Cen Qian, Li-Shang Dai, Yu Sun, Muhammad Nadeem Abbas, Bao-Jian Zhu, Chao-Liang Liu

**Affiliations:** 1 College of Life Sciences, Anhui Agricultural University, 130 Changjiang West Road, Hefei 230036, Anhui Province, P. R. China

## Abstract

The complete mitochondrial genome (mitogenome) of *Leucoma salicis* (Lepidoptera: Lymantriidae) was sequenced and annotated. It is a circular molecule of 15,334 bp, containing the 37 genes usually present in insect mitogenomes. All protein-coding genes (PCGs) are initiated by ATN codons, other than *cox1*, which is initiated by CGA. Three of the 13 PCGs had an incomplete termination codon, T or TA, while the others terminated with TAA. The relative synonymous codon usage of the 13 protein-coding genes (PCGs) was consistent with those of published lepidopteran sequences. All tRNA genes had typical clover-leaf secondary structures, except for the *tRNA*^*Ser*^ *(AGN)*, in which the dihydrouridine (DHU) arm could not form a stable stem-loop structure. The A + T-rich region of 325 bp had several distinctive features, including the motif ‘ATAGA’ followed by an 18 bp poly-T stretch, a microsatellite-like (AT)_7_ element, and an 11-bp poly-A present immediately upstream of *tRNA*^*Met*^. Relationships among 32 insect species were determined using Maximum Likelihood (ML), Neighbor Joining (NJ) and Bayesian Inference (BI) phylogenetic methods. These analyses confirm that *L. salicis* belongs to the Lymantriidae; and that Lymantriidae is a member of Noctuoidea, and is a sister taxon to Erebidae, Nolidae and Noctuidae, most closely related to Erebidae.

*Leucoma salicis* is a moth that is mainly distributed in China, Korea and Japan. It is a notorious plant pest and causes considerable economic losses. It typically consumes willow and tea leaves, influencing quality and quantity of tea products^1^; and damages roadside and garden trees in urban areas. Traditionally, the identification of this species was based on morphological characteristics of adult moths^2^. However, the moth appears mainly in June to August, the rest of its life go through egg and larva stages (which has no easily identifying morphological features), requiring eggs and larvae to be reared to adult stage for identification, which is time consuming and labor intensive. Molecular methods for identification are under development, including polymerase chain reaction-restriction fragment length polymorphism (PCR–RFLP)^3^. Most previous work on *L. salicis* has focused on sex pheromone synthesis^4^, or the nuclear polyhedrosis virus that infects larvae^5^. Previous studies have not focused on the mitochondrial genome, which can provide systematically-informative information for identification, phylogenetic analysis and evolutionary studies on *L. salicis*.

Insect mitochondrial DNA (mtDNA) is a double-stranded, circular molecule, ranging in size from 14 to 20 kb. It usually contains a conserved set of 37 genes, including seven *NADH dehydrogenase* (*nad1-nad6* and *nad4L*), three *cytochrome c oxidase* (*cox1-cox3*), two *ATPase* (*atp6 and atp8*), one *cytochrome b* (*cob*), two ribosomal RNA (*rrnL and rrnS*), 22 transfer RNA (tRNA) genes, and an adenine (A) + thymine (T)-rich region that contains initiation sites for transcription and replication of the genome^6^,^7^. Due to its simple genomic organization, high rate of evolution, and almost unambiguous orthology, mtDNA is typically considered to be an informative molecular marker for species identification and in studies of phylogenetic relationships and population structure^8^,^9^.

A better understanding of the lepidopteran mitochondrial genome requires expanded taxon sampling. Lepidoptera contains more than 160,000 described species, classified into 45–48 superfamilies^10^. Lymantriidae includes about 360 genera and over 2500 species, many of which are agriculturally important. Only eight species have completely-sequenced mitogenomes that are publically available in GenBank, despite the large species diversity in the family. In this study, we sequenced and annotated the complete mitogenome sequence of *L. salicis,* and compared it with those of other members of Lymantriidae. Our results provide novel methods for species identification of an important pest, as well as phylogenetically-informative sequence data that addresses the position of *L. salicis* within Noctuoidea.

## Results

### Geno me organization and composition

**Figure 1:**
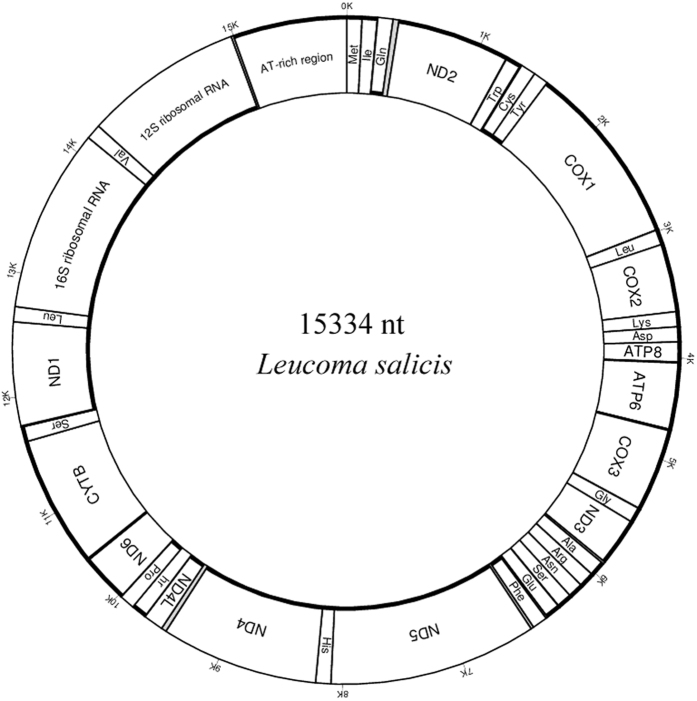
Map of the mitogenome of *L. salicis.* tRNA genes are labeled according to the IUPAC-IUB three-letter amino acids; *cox1, cox2* and *cox3* refer to the cytochrome c oxidase subunits; *cob* refers to cytochrome b; *nad1-nad6* refer to NADH dehydrogenase components; *rrnL* and *rrnS* refer to ribosomal RNAs.

The mitogenome of *L. salicis* was a circular DNA molecule, 15,334 bp in length (Fig. 1). It contained the typical insect mitogenome set of 22 tRNAs, 13 PCGs (*nad1-6, nad4L, cox1-3, cob, atp6* and *atp8*), two rRNAs (*rrnS and rrnL*), and the non-coding A + T-rich region (Table 1). Nucleotide composition was highly A + T biased (A: 42.07%, T: 38.57%, G: 7.22%, C: 12.14%; Table 2). Nucleotide BLAST (blastn) of the entire *L. salicis* mitogenome against GenBank returned sequence identities with closely related species of 79% (*Lachana alpherakii*), 78% (*Euproctis pseudoconspersa*), 78% (*Gynaephora menyuanensis*), and 77% (*Lymantria dispar*) (Table S1).

### Protein-coding genes and codon usage

The PCG region formed 72.9% of the *L. salicis* mitogenome, and was 11,172 bp long. Nine of 13 PCGs (*nad2, cox1, cox2, atp8, atp6, cox3, nad3, nad6* and *cob*) were encoded on the H-strand, while the remaining four (*nad5, nad4, nad4L* and *nad1*) were encoded on the L-strand. Each PCG was initiated by a canonical ATN codon, except for *cox1* (Table 1), which was initiated by a CGA codon. Ten of 13 PCGs used a typical TAA termination codon; but *cox1* and *cox2* terminated with a single T and *nad4* terminated with TA (Table 1).

**Figure 2:**
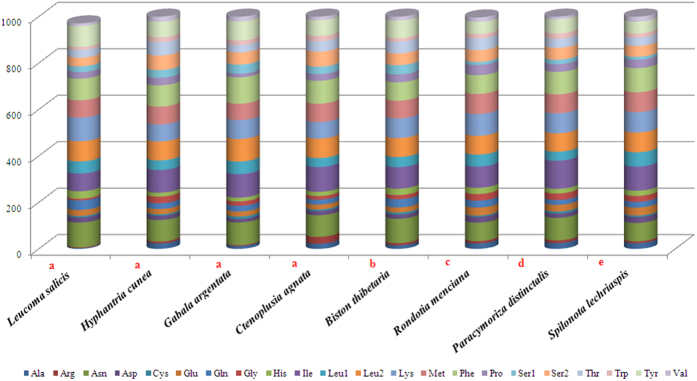
Comparison of codon usage within the mitochondrial genome of members of the Lepidoptera. Lowercase letters (**a,b,c,d and e**) above species names represent the superfamily to which the species belongs (a: Noctuoidea, b: Geometroidea, c: Bombycoidea, d: Pyraloidea, e: Tortricoidea).

**Figure 3:**
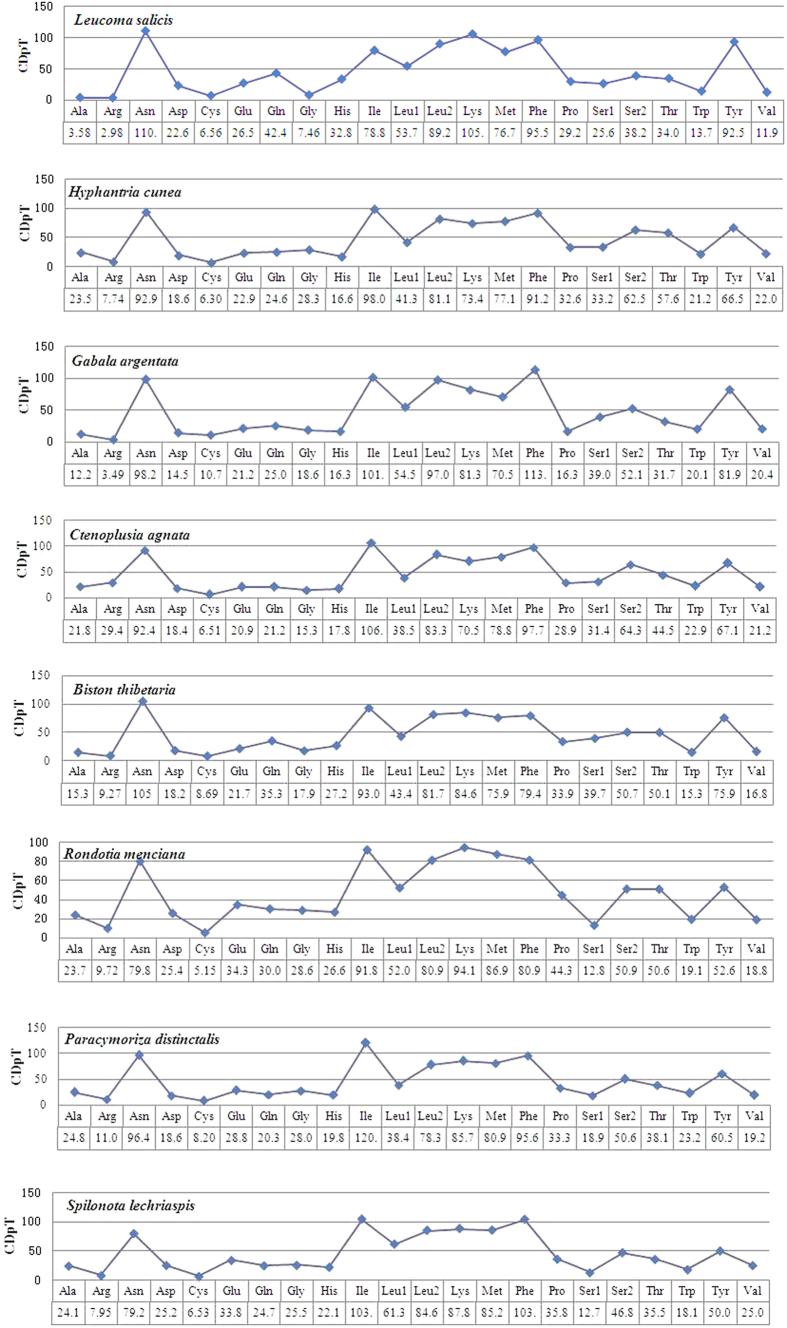
Codon distribution in members of the Lepidoptera. CDspT = codons per thousand codons.

**Figure 4:**
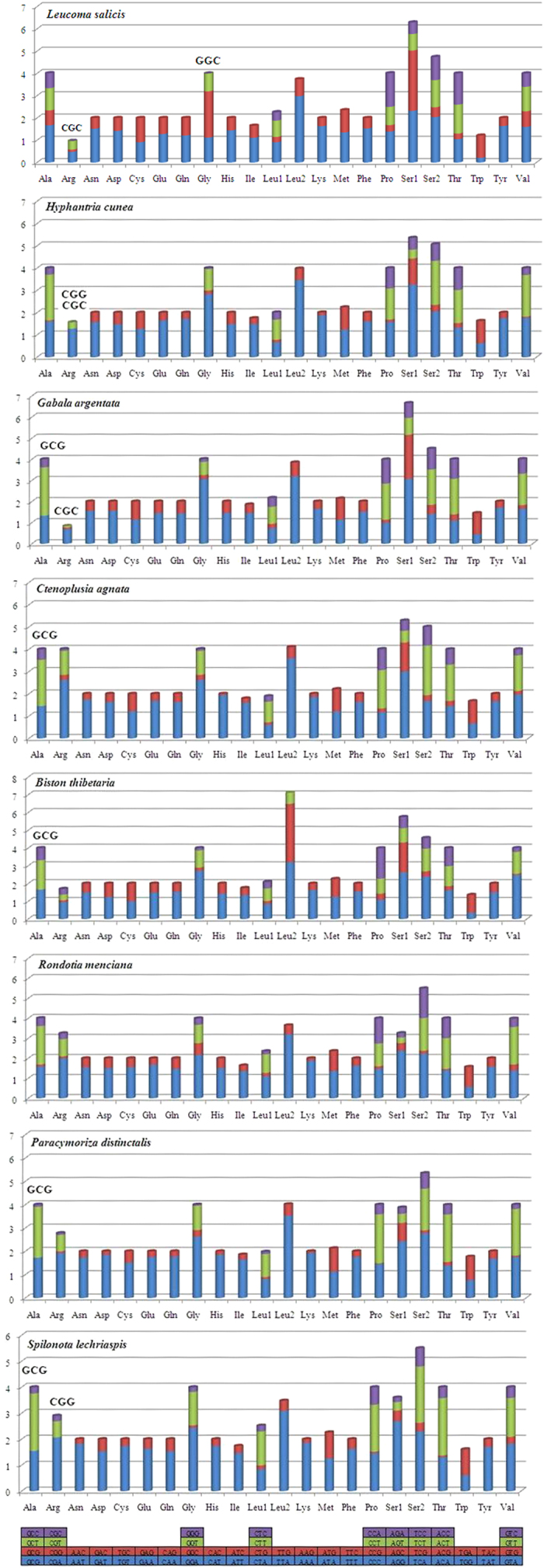
Relative Synonymous Codon Usage (RSCU) of the mitochondrial genome of five superfamilies in the Lepidoptera. Codon families are plotted on the x-axis. Codons indicated above the bar are not present in the mitogenome.

Relative synonymous codon usage (RSCU) analysis of PCGs in *L. salicis* revealed that the codons encoding Asn, Ile, Leu (UUA, UUG), Lys, Tyr and Phe were the most frequently present, while those encoding Cys and Arg were rare (Fig. 2). In the PCGs of the eight moth species examined, codon distributions and amino acid content were largely consistent among species (Fig. 3). Codons with A or T in the third position were overused in comparison to other synonymous codons: for example, the codons for valine GTC and GTG were rare, while the synonymous codons GTT and GTA were prevalent (Fig. 4). All used codons were present in the PCGs of the *L. salicis* mitogenome, except for CGC and GGC. This is similar to codon usage in *Hyphantria cunea, Spilonota lechriaspis*, and *Gabala argentata*, which respectively lack CGG and CGC, GCG and CGG, and CGG and CGC.

### Ribosomal RNA and transfer RNA genes

The large (*rrnL*) and small (*rrnS*) ribosomal RNA subunit genes of *L. salicis* were located between the *tRNA*^*Leu1*^*(CUN)*/*tRNA*^*Val*^ and the *tRNA*^*Val*^/A + Trich regions, respectively (Fig. 1, Table 1). The *rrnL* gene was 1,344 bp long, while *rrnS* was 840 bp long. A + T content of the rRNA genes was 83.91%. AT and GC skews were positive (0.029) and negative (**−**0.144), respectively.

**Figure 5:**
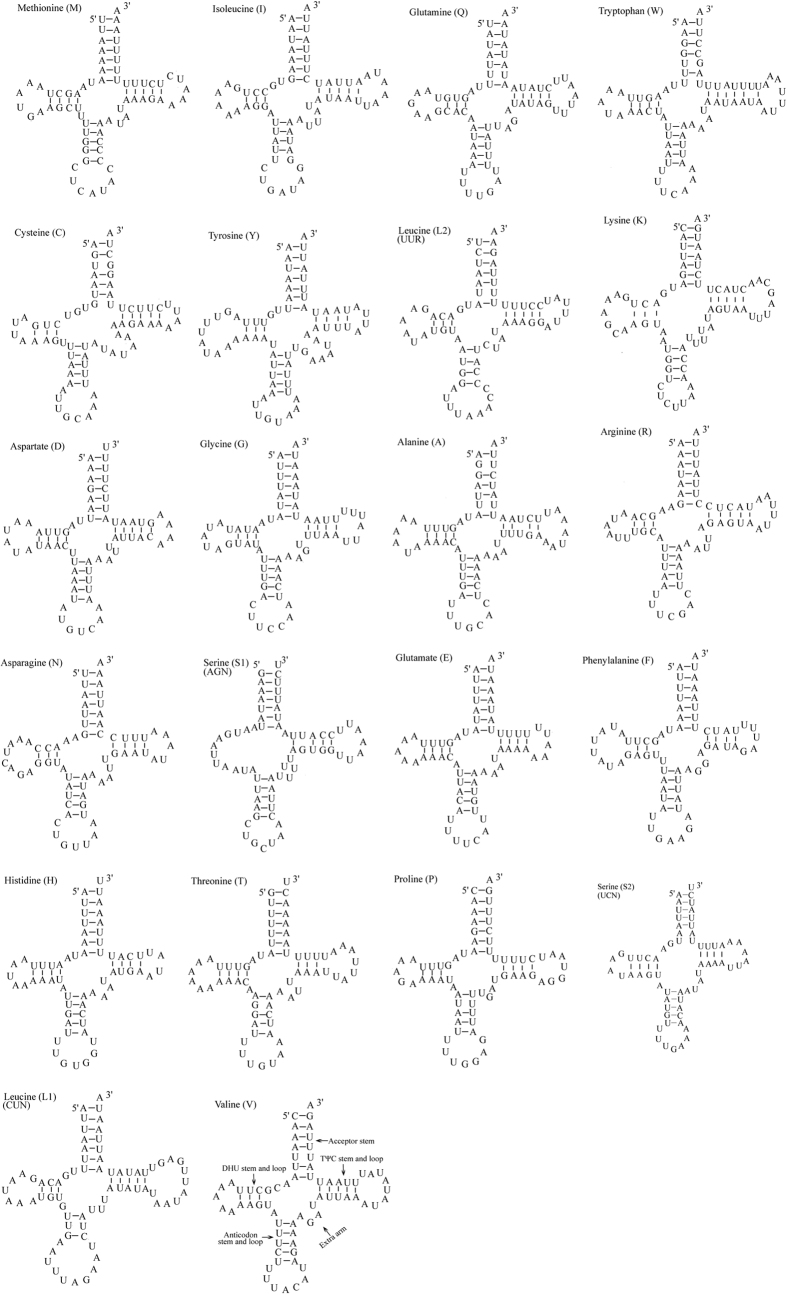
Predicted secondary structures of the 22 tRNA genes of the *L. salicis* mitogenome.

The *L. salicis* mitogenome included 22 tRNA genes, ranging from 64 bp (*tRNA*^*His*^) to 73 bp (*tRNA*^*Trp*^) long. Of these, 14 genes were encoded on the H-strand and eight on the L-strand (Table 1). The tRNA genes were highly A + T biased (82.19%) with a positive AT-skew (0.007) (Table 2). All the tRNAs possessed a typical clover-leaf secondary structure, except *tRNA*^*Ser*^*(AGN)*, which lacks the dihydrouridine (DHU) arm and forms a simple loop (Fig. 5). Ten of the tRNA genes were each found to have 11 G-U mismatches in their respective secondary structures, which form a weak bond. Ten U-U mismatches were present in the respective amino acid acceptor stems of *tRNA*^*Gln*^, *tRNA*^*Trp*^, *tRNA*^*Leu*^*(UUR), tRNA*^*Ala*^, *tRNA*^*Thr*^, *tRNA*^*Leu*^*(CUN)*, and *tRNA*^*Val*^ (Fig. 5). All tRNA secondary structures of the tRNA genes were calculated using the tRNAscan-SE program.

### Overlapping and intergenic spacer regions

We identified four overlapping gene sequences, varying from 1 bp to 8 bp, making up 19 bp in total. The longest overlapping region was 8 bp between *tRNA*^*Trp*^ and *tRNA*^*Cys*^; there was a 7 bp overlap between *atp8* and *atp6*; 3 bp overlap between *tRNA*^*Ile*^ and *tRNA*^*Gln*^, and 1 bp between *tRNA*^*Ala*^ and *tRNA*^*Arg*^ (Table 1).

**Figure 6:**
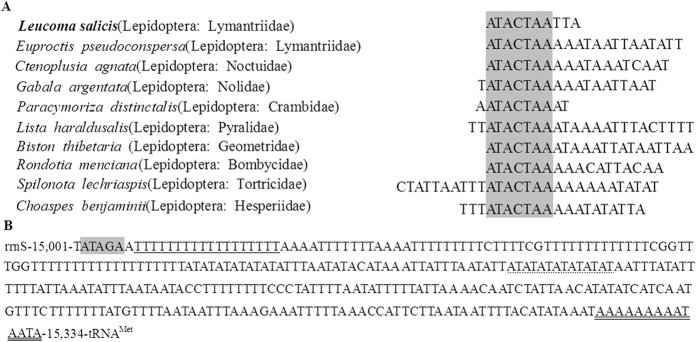
(**A**) Alignment of the intergenic spacer region between *tRNA*^*Ser*^ *(UCN)* and *nad1* of several Lepidopteran insects. (**B**) Features present in the A + T-rich region of *L. salicis.* The ‘ATATG’ motif is shaded. The poly-A stretch is double underlined, and the poly-T stretch is underlined. The single microsatellite T/A repeat sequence is indicated by dotted underlining.

Intergenic spacers were spread over 18 regions, and ranged in length from 1 bp to 47 bp. The longest (47 bp) contained an A + T-rich region and occurred between *tRNA*^*Gln*^ and *nad2*. The 10 bp spacer region between *tRNA*^*Ser*^ *(UCN)* and *nad1* included an ‘ATACTAA’ motif (Fig. 6A).

### The A + T-rich region

The 325 bp long A + T-rich region of *L. salicis* was located between the *rrnS* and *tRNA*^*Met*^ genes (Table 1). A + T content in the A + T-rich region was 91.69%, and both AT (−0.248) and GC (−0.408) skews were negative (Table 2). The A + T-rich region did not contain long repeats, though some short repeating sequences scattered over the entire region were present: an ‘ATAGA’ motif followed by an 18 bp poly-T stretch, a microsatellite-like (AT)_7_ and a poly-A element upstream of the *tRNA*^*Met*^ gene (Fig. 6B).

### Phylogenetic relationships

**Figure 7:**
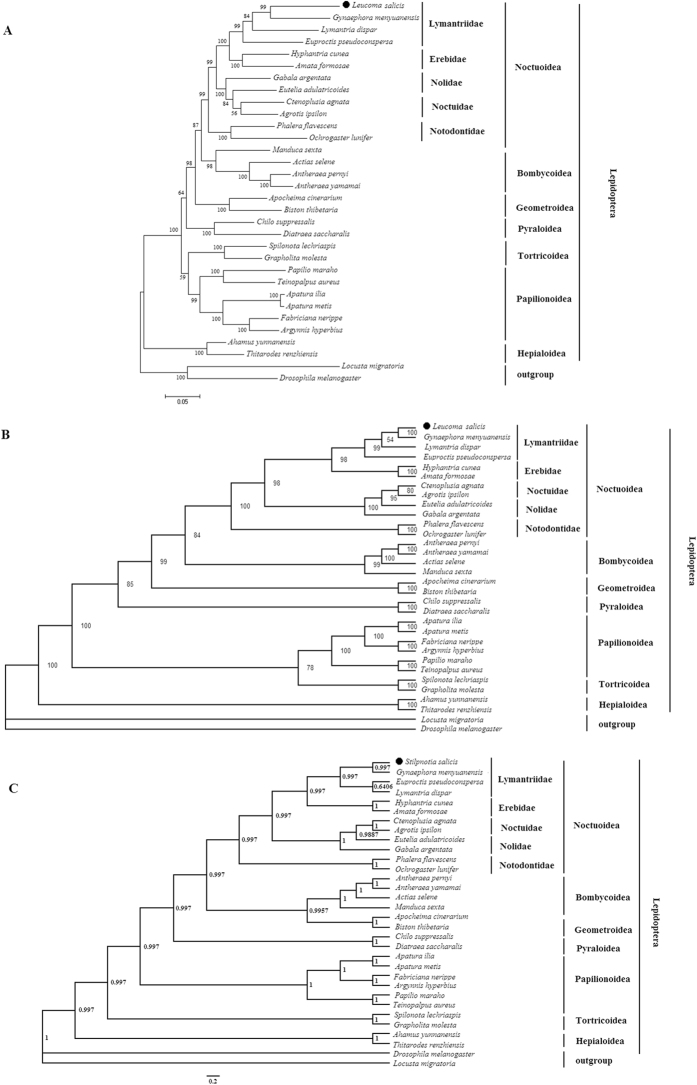
(**A**) Tree showing the phylogenetic relationships among 32 species, constructed using Maximum Likelihood with 1000 bootstrap replicates. (**B**) Neighbor Joining (NJ) tree, with 1000 bootstrap replicates. (**C**) Tree constructed using Bayesian Inference (BI) MCMC consensus tree, with posterior probabilities shown at nodes. *Drosophila melanogaster* (NC_025936) and *Locusta migratoria* (NC_002084) were used as outgroups.

We established phylogenetic relationships among 32 insects (Table 3), based on nucleotide sequences of 13 PCGs, using Maximum Likelihood (ML), Neighbor Joining (NJ) and Bayesian Inference (BI) methods. Species clustered by family (Fig. 7A, B and C). Within Lymantriidae, *L. salicis* was most closely related to *G. menyuanensis*. Lymantriidae clustered with Erebidae, while Noctuidae clustered with Nolidae. Noctuoidea was most closely related to Bombycoidea in ML and NJ trees, while in the BI tree Bombycoidea was most closely related to Geometroidea. Papilionoidea and Tortricoidea branched together in ML and NJ methods, but were separated from each other in the BI tree.

## Discussion

At the family level, the length of the *L. salicis* mitogenome (15,334 bp) is marginally smaller than that of *Euproctis pseudoconspersa* (15,461 bp), but it falls within the range (15,140–16,173 bp) of other known lepidopteran mitogenomes. Gene order and orientation are the same as in previously-sequenced Lymantriidae. Nucleotide BLAST (blastn) result of the entire mitogenome against closely related species revealed that *L. salicis* has a high similarity with the Lymantriidae species (77% in *L. dispar*–79% in *L. alpherakii*). The conserved regions lie in 22 tRNAs and 13 PCGs, while A + T-rich region varies in these species. These remarkable characteristics have been reported in other lepidopteran species^7^ and could be used as potential markers for identification at genus and species level in recent molarcular techniques. The highly A + T biased nucleotide composition is within the range of previously sequenced lepidopterans (79.64% in *L. dispar*–81.48% in *G. menyuanensis*). The positive AT skew (0.043) observed here, indicating the presence of more As than Ts, is similar to that seen in many lepidopterans, including *L. dispar* (0.014), *Rondotia menciana* (0.050), and *Biston thibetaria* (0.064) (Table 2). It is slightly higher than that of other sequenced mitogenomes in Noctuoidea, including *Ctenoplusia agnata* (−0.023), *G. menyuanensis* (0.003) and *E. pseudoconspersa* (0.011). A similar trend has been observed in other lepidopteran superfamilies such as Bombycoidea, where AT skew varies from 0.001 (*Sphinx morio*) to 0.059 (*Bombyx mori*)^11^. In all sequenced lepidopteran mitogenomes, GC skew ranges from −0.268 in *G. menyuanensis* to −0.155 in *Paracymoriza distinctalis* (Table 2). The *L. salicis* mitogenome is moderately skewed (−0.254), showing the presence of more Cs than Gs.

The AT skew value (0.063) of the protein-coding gene region in the *L. salicis* mitogenome is higher than that of several previously sequenced mitogenomes. Its negative GC skew (**−**0.234) is similar to that seen in other animals. *Cox1* is thought to initiate with CGA, as found in other lepidopteran insects^12^,^13^. *Cox1* and *cox2* terminate with a single T, while *nad4* terminates with TA. Similar results have been documented in several sequenced lepidopteran mitogenomes, including *Artogeia melete*^14^, *Phthonandria atrilineata*^15^, *Ochrogaster lunifer*^16^, *H. cunea*^17^ and *Amata emma*^18^. The common termination codon TAA is usually created via post-transcriptional polyadenylation^19^. The relative synonymous codon usage of the 13 protein-coding genes (PCGs) in *L. salicis* is consistent with those of published lepidopteran sequences. Similarly, codons with A or T in the third codon position being overrepresented relative to other synonymous codons, is consistent with previous observations of lepidopterans^9^; likewise the absence or underrepresentation of high-GC codons^18^,^20^.

The A + T content (83.91%) of rRNA genes is similar to that seen in Lymantriidae (83.05% in *G. menyuanensis*). The positive AT (0.029) and negative GC (**−**0.144) skew seen in the *L. salicis* mitogenome has also been reported in several sequenced lepidopterans (Table 2). For example, *H. cunea* has a positive AT (0.024) and negative GC (**−**0.137) skew^17^; and *L. dispar* also has positive AT (0.023) and negative GC (**−**0.155) skew.

The secondary structure of *L. salicis tRNA*^*Ser*^*(AGN)* lacks the dihydrouridine (DHU) arm and forms a simple loop. This has also been observed in several other animal mitogenomes^21^, including those of insects^15^,^22^,^23^. Ten tRNA genes have 11 mismatches in their secondary structures; most of these are located in the acceptor, DHU and anticodon stems. In addition, *tRNA*^*Cys*^ and *tRNA*^*Ser*^ *(UCN)* contain an A-A mismatch in the anticodon stem. Unmatched base pairs observed in tRNA sequences can be corrected by RNA-editing mechanisms that are well known for arthropod mtDNA^24^.

Four overlapping sequences occur in the mitogenome of *L. Salicis*. The 7 bp overlap between *atp8* and *atp6* has been documented in several other lepidopteran mitogenomes^25^,^26^. The 10 bp intergenic spacer region containing an ‘ATACTAA’ motif, between *tRNA*^*Ser*^ *(UCN)* and *nad1*, has also been documented in at least nine other species, suggesting that this region is highly conserved among most of the lepidopteran mtDNAs sequenced to date^27^.

The length of the A + T-rich region of *L. salicis* (325 bp) is shorter than those of *G. menyuanensis* (449), *L. dispar* (371), *H. cunea* (357) and *B. thibetaria* (350), and longer than those of *Lista haraldusalis* (310) and *Choaspes benjaminii* (293). Extra tRNA-like structures are often found in the A + T-rich region of lepidopteran mitogenomes. For example *Antheraea yamamai* has *tRNA*^*Ser*^*(UCN)*-like and *tRNA*^*Phe*^-like sequences, each with correct anticodon structure and forming a clover-leaf structure, which suggests that they may be functional, though each has several mismatches in both aminoacyl and anticodon stem regions^28^. Extra tRNA-like structures have not been seen in *L. salicis*. The presence of multiple tandem-repeat elements is described as being characteristic of insect A + T-rich regions^29^. *Antheraea pernyi* has a repeat element of 38 bp tandemly repeated six times^25^; and *Cnaphalocrocis medinalis* has a duplicated 25 bp repeat element^25^,^30^. Long conspicuous repeats were not observed in the A + T-rich region of *L. salicis*, though shorter repeating sequences, an ‘ATAGA’ motif and other features were. These characteristic features have each been found in previously sequenced lepidopteran species^27^,^31^,^32^.

In general, the *L. salicis* mitogenome contains several features in nucleotide composition, structure of tRNAs and PCGs as well as in the A + T rich region. Particularly in advanced technologies like PCR–RFLP methods^3^ and DNA barcodes^33^, these similarities and differences between *L. salicis* and other insects could be used as potential markers in species identification, especially the differences.

Phylogenetic relationships were established using Maximum Likelihood (ML) Neighbor Joining (NJ) and Bayesian Inference (BI) methods. Species clustered in families, and results were broadly consistent with previous work, *e.g.* Dong *et al*.^26^ and Dai *et al*.^34^. Results obtained from our analyses also supported the classification proposed by Fibiger and Lafontaine^35^, including within Lymantriidae a clade comprised of *E. pseudoconspersa, L. salicis, L. dispar* and *G. menyuanensis*. The present analysis showed that within Lymantriidae, *L. salicis* was most closely related to *G. menyuanensis*, which is consistent with a recent study on *E. pseudoconspersa*^26^. Interestingly, *L. dispar* is more closely related to *G. menyuanensis* than *E. pseudoconspersa* in ML and NJ trees (Fig. 7A and B), whereas in the BI consensus tree *L. dispar* and *E. pseudoconspersa* branch together with 0.6406 posterior probabilities (Fig. 7C). We conclude from the above results that differences between BI, ML and NJ methods generate different results on the relationship among different Noctuoidea species.

Because most previous classifications of Lymantriidae species have been based on morphological features, the precise position of Lymantriidae within the Noctuoidea is still unclear. Kitching has suggested that the Lymantriidae are the sister group to a paraphyletic Pantheidae, sharing apomorphies such as the presence of secondary setae in first instar larvae^36^. Zahiri *et al*. reclassified the Noctuoidea on the basis of molecular analyses, making the group currently named Lymantriinae a subfamily of Erebidae^37^. Our results suggest that Lymantriidae can be regarded as a sister group to other families (Erebidae, Nolidae and Noctuidae) in the Noctuoidea, being most closely related to Erebidae that is consistent with previous study of Fibiger and Lafontaine (2005) on higher Noctuoidea classification. They placed the Lymantriidae from a position in front of the Nolidae to a position after Arctiidae to reflect the close association of the arctiids and lymantriids, and moved the Nolidae, Arctiidae and Lymantriidae in front of the upgraded family Erebidae so that their close relationship with the “quadrifids’ is better reflected^35^. It is concluded that further studies are needed on sequencing and characterization of mitogenomes of the family Lymantriidae that will provide insight to classification of Noctuoidea.

At the level of superfamilies, Noctuoidea was closely related to Bombycoidea in our ML and NJ analyses, while in the BI tree, Bombycoidea was closely related to Geometroidea. Papilionoidea and Tortricoidea branched together in ML and NJ trees, but in the BI tree they formed separate branches, more in line with previous studies. Hepialoidea was the sister group to all other superfamilies, as found previously by Salvato *et al*.^16^ and Chai *et al*.^38^. While several previous studies have been undertaken on mitogenomes of Noctuoidea, relatively little is known about Lymantriidae specifically. Further taxon sampling within Lymantriidae and related families is required to resolve the placement of Lymantriidae in Noctuoidea.

## Materials and Methods

### Sample collection and mitochondrial DNA extraction

*L. salicis* larvae were collected from willow trees within the campus of Anhui Agricultural University, Hefei, China. Total genomic DNA was extracted using the Aidlab Genomic DNA Extraction Kit (Aidlab Co., Beijing, China) according to the manufacturer’s instructions. Quality of extracted DNA was assessed by electrophoresis on a 1% agarose gel stained with ethidium bromide.

### Primer design, PCR amplification and sequencing

The full mitochondrial genome of *L. salicis* was PCR amplified in thirteen overlapping fragments, based on primers that were designed from known mitogenomes of Lymantriidae, and synthesized by Invitrogen Co. Ltd. Shanghai, China (Table 4). All PCRs were performed in a 50 μL reaction volume, including 35 μL sterilized distilled water, 5 μL 10 × Taq buffer (Mg^2 + ^), 4 μL dNTP (25 mM), 1.5 μL DNA, 2 μL of each primer (10 μM) and 0.5 μL (1 unit) Taq (TaKaRa Co., Dalian, China). PCR conditions were as follows: 4 min at 94 °C, followed by 35 cycles of 30 s at 94 °C, 40 s at 46–58 °C (Table 4), and 1–3 min (depending on putative length of the fragments) at 72 °C; and then a final extension step of 72 °C for 10 min.

All PCR products were visualized by electrophoresis on a 1.0% TAE agarose gel, and purified using a DNA gel extraction kit (Transgen Co., Beijing, China). The purified PCR fragments were ligated into the T-vector (TaKaRa Co., Dalian, China) and transformed into *Escherichia coli* DH5α, using the manufacturer’s protocol. Recombinants were cultured overnight at 37 °C on Luria-Bertani (LB) solid medium containing Ampicillin (AMP), isopropylthiogalactoside (IPTG) and 5-bromo-4-chloro-3-indolyl-D-galactopyranoside (X-Gal). White colonies carrying insert DNA were selected, cultured overnight in liquid media, and vector inserts were directly sequenced by Sangon Biotech Co., (Shanghai, China).

### Sequence assembly and gene annotation

The complete mtDNA sequence was assembled using the SeqManII program from the Lasergene software package (DNAStar Inc., Madison, USA). Sequence annotation was performed using the NCBI’s web interface for BLAST (http://blast.ncbi.nlm.nih.gov/Blast).

Nucleotide sequences of the PCGs were translated into putative proteins based on insect sequences available in GenBank. Initiation and termination codons were identified using an alignment created in ClustalX version 2.0, with other lepidopteran sequences as references. To describe base composition, we analyzed skew as described by Junqueira^39^: AT skew = [A − T]/[A + T], GC skew = [G − C]/[G + C]. The relative synonymous codon usage (RSCU) was obtained using MEGA 5^40^.

The tRNA genes were verified using the program tRNAscan-SE with default settings^41^, in addition to using the alignment to visually identify sequences with the appropriate anticodons capable of folding into the typical clover-leaf secondary structure. In the A + T-rich region, tandem repeats were found with the Tandem Repeats Finder program (http://tandem.bu.edu/trf/trf.html)^42^.

### Phylogenetic analysis

A total of 29 sets of 13 PCG sequences were used to perform phylogenetic analysis, including those of *L. salicis.* Those from other taxa were downloaded from GenBank, with *Drosophila melanogaster* (U37541.1)^43^ and *Locusta migratoria* (JN858212)^44^ sequences used as an outgroup. Alignments of the 13 concatenated PCGs were conducted using ClustalX version 2.0. Maximum likelihood (ML) phylogenetic analysis was performed using MEGA 5.0 with Tamura-Nei model^40^. Neighbor Joining (NJ) distance analysis was performed using PAUP4b10^45^, and Bayesian Inference (BI) MCMC phylogenetic analysis was performed using MrBayes 3.2^46^. The ML analysis was pseudosampled with 1000 bootstrapped datasets. The NJ analysis was done with 1000 bootstrap replicates. The BI analysis used four chains MCMC, running for 1,000,000 generations, with trees being sampled every 1000 generations. The consensus tree was visualized using FigTree v1.4.0 (http://tree.bio.ed.ac.uk/software/figtree/).

## Additional Information

**How to cite this article**: Sun, Y.-X. *et al*. Characterization of the Complete Mitochondrial Genome of *Leucoma salicis* (Lepidoptera: Lymantriidae) and Comparison with Other Lepidopteran Insects. *Sci. Rep.* **6**, 39153; doi: 10.1038/srep39153 (2016).

**Publisher's note:** Springer Nature remains neutral with regard to jurisdictional claims in published maps and institutional affiliations.

## Supplementary Material

Supplementary Table S1

## Figures and Tables

**Table 1:** Summary of characteristics of the mitogenome of *L. salicis*.

Gene	Direction	Location	Size	Anti codon	Start codon	Stop codon	Intergenic Nucleotides
tRNAMet	F	1–66	66	CAT	—	—	0
tRNAIle	F	67–134	68	GAT	—	—	−3
tRNAGln	R	132–200	69	TTG	—	—	47
ND2	F	248–1233	986	—	ATT	TAA	3
tRNATrp	F	1237–1309	73	TCA	—	—	−8
tRNACys	R	1302–1370	69	GCA	—	—	0
tRNATyr	R	1371–1442	72	GTA	—	—	1
COI	F	1453–2987	1535	—	CGA	T	2
tRNALeu(UUR)	F	2993–3059	67	TAA	—	—	0
COII	F	3060–3740	681	—	ATT	T	0
tRNALys	F	3741–3811	71	CTT	—	—	0
tRNAAsp	F	3812–3878	67	GTC	—	—	0
ATP8	F	3879–4040	162	—	ATA	TAA	−7
ATP6	F	4034–4711	678	—	ATG	TAA	14
COIII	F	4726–5514	789	—	ATG	TAA	2
tRNAGly	F	5517–5582	66	TCC	—	—	0
ND3	F	5583–59336	354	—	ATT	TAA	10
tRNAAla	F	5947–6017	71	TGC	—	—	−1
tRNAArg	F	6017–6084	68	TCG	—	—	1
tRNAAsn	F	6086–6152	67	GTT	—	—	0
tRNASer(AGN)	F	6153–6219	67	GCT	—	—	17
tRNAGlu	F	6237–6301	65	TTC	—	—	0
tRNAPhe	R	6302–6368	67	GAA	—	—	20
ND5	R	6389–8103	1715	—	—	—	8
tRNAHis	R	8112–8175	64	GTG	—	—	1
ND4	R	8177–9516	1341	—	ATG	TA	15
ND4L	R	9532–9825	294	—	—	—	5
tRNAThr	F	9831–9897	67	TGT	—	—	0
tRNAPro	R	9898–9965	68	TGG	—	—	2
ND6	F	9968–10514	547	—	ATA	TAA	8
Cytb	F	10523–11655	1133	—	ATG	TAA	1
tRNASer(UCN)	F	11657–11721	65	TGA	—	—	10
ND1	R	11732–12684	953	—	ATT	TAA	0
tRNALeu(CUN)	R	12685–12755	71	TAG	—	—	0
lrRNA	R	12756–14099	1344	—	—	—	0
tRNAVal	R	14100–14169	70	TAC	—	—	0
srRNA	R	14170–15009	840	—	—	—	0
A + T-rich region	—	15010–15334	325	—	—	—	—

**Table 2:** Composition and skew in different lepidopteran mitogenomes.

Species	Size (bp)	A%	G%	T%	C%	A + T %	AT skewness	GC skewness
Whole genome								
* L. salicis*	15334	42.07	7.22	38.57	12.14	80.64	0.043	−0.254
* C. agnata*	15261	39.58	7.71	41.52	11.2	81.1	−0.023	−0.184
* H. cunea*	15481	40.58	7.55	39.81	12.06	80.39	0.009	−0.229
* G. menyuanensis*	15770	40.88	6.77	40.6	11.75	81.48	0.003	−0.268
* L. dispar*	15507	40.38	7.61	39.26	12.5	79.64	0.014	−0.243
* E. pseudoconspersa*	15461	40.42	7.61	39.51	12.46	79.93	0.011	−0.241
* G. argentata*	15337	39.64	7.56	42.05	10.75	81.69	−0.029	−0.174
* A. formosae*	15463	38.67	7.53	40.83	12.98	79.49	−0.027	−0.265
* P. distinctalis*	15354	41.04	7.49	41.22	10.24	82.27	−0.002	−0.155
* L. haraldusalis*	15213	40.47	7.66	41.04	10.83	81.52	−0.006	−0.171
* B. thibetaria*	15484	42.38	7.55	37.24	12.83	79.62	0.064	−0.259
* R. menciana*	15301	41.42	7.82	37.45	13.31	78.86	0.050	−0.259
* B. mori*	15666	43.09	7.31	38.26	11.34	81.35	0.059	−0.216
* S. morio*	15299	40.64	7.58	40.53	11.26	81.17	0.0013	−0.195
* S. lechriaspis*	15368	39.86	7.63	41.34	11.17	81.19	−0.018	−0.188
* C. benjaminii*	15272	40.08	7.52	40.7	11.7	80.78	−0.007	−0.217
PCG								
* L. salicis*	11171	42.24	7.89	37.16	12.71	79.39	0.063	−0.233
* C. agnata*	11238	39.12	8.37	40.79	11.72	79.91	−0.020	−0.166
* H. cunea*	11205	39.99	8.35	38.6	13.06	78.59	0.017	−0.219
* G. menyuanensis*	11228	40.37	7.5	39.41	12.72	79.78	0.012	−0.258
* L. dispar*	11236	39.52	8.44	38.18	13.62	77.71	0.017	−0.234
* E. pseudoconspersa*	11187	3969	8.43	38.3	13.58	77.99	0.017	−0.233
* G. argentata*	11203	39.05	8.29	41.27	11.38	80.33	−0.027	−0.157
* A. formosae*	11,217	38.18	8.28	39.62	13.92	77.8	−0.018	−0.254
* P. distinctalis*	11189	40.54	8.12	40.53	10.81	81.07	0	−0.142
* L. haraldusalis*	11,193	39.88	8.47	40.16	11.49	80.04	−0.003	−0.151
* B. thibetaria*	11212	41.66	8.36	35.94	14.04	77.6	0.073	−0.253
* R. menciana*	11225	40.97	8.58	36.12	14.33	77.1	0.063	−0.251
* B. mori*	11177	42.93	8.16	36.64	12.28	79.57	0.079	−0.201
* S. morio*	11179	40.28	8.27	39.56	11.89	79.84	0.009	−0.179
* S. lechriaspis*	11258	39.31	8.35	40.41	11.93	79.72	−0.013	−0.176
* C. benjaminii*	11153	39.44	8.23	39.74	12.59	79.18	−0.003	−0.209
tRNA								
* L. salicis*	1498	43.19	6.88	40.72	9.21	83.91	0.029	−0.145
* C. agnata*	1477	41.23	8.19	40.22	10.36	81.45	0.012	−0.117
* H. cunea*	1474	41.86	7.87	39.89	10.38	81.75	0.024	−0.138
* G. menyuanensis*	1504	41.29	7.38	41.76	9.57	83.05	−0.006	−0.129
* L. dispar*	1466	41.41	7.98	39.5	10.91	80.9	0.024	−0.155
* E. pseudoconspersa*	1466	41.41	8.19	40.18	10.23	81.58	0.015	−0.111
* G. argentata*	1469	41.32	8.24	40.23	10.21	81.55	0.013	−0.107
* A. formosae*	1457	40.43	7.96	40.36	11.26	80.78	0.001	−0.172
* P. distinctalis*	1536	42.19	8.14	39.78	9.9	81.97	0.029	−0.098
* L. haraldusalis*	1451	41.08	7.86	41.42	9.65	82.49	−0.004	−0.102
* B. thibetaria*	1478	42.08	7.85	39.24	10.83	81.33	0.035	−0.160
* R. menciana*	1485	41.08	8.08	39.93	10.91	81.01	0.014	−0.149
* B. mori*	1470	42.04	7.89	39.52	10.54	81.56	0.031	−0.144
* S. morio*	1463	40.6	8.2	41.01	10.18	81.61	−0.005	−0.108
* S. lechriaspis*	1516	41.03	7.92	41.09	9.96	82.12	−0.001	−0.114
* C. benjaminii*	1467	40.9	8.04	40.49	10.57	81.39	0.005	−0.136
rRNA								
* L. salicis*	2184	41.39	5.04	40.8	12.77	82.19	0.007	−0.434
* C. agnata*	2112	40.01	5.07	44.65	10.27	84.66	−0.055	−0.339
* H. cunea*	2234	42.08	4.92	42.75	10.25	84.83	−0.008	−0.351
* G. menyuanensis*	2311	41.89	4.28	42.84	10.99	84.73	−0.011	−0.439
* L. dispar*	2140	42.52	4.81	41.82	10.42	84.35	0.008	−0.368
* E. pseudoconspersa*	2225	42.56	4.54	42.11	10.79	84.67	0.005	−0.408
* G. argentata*	2165	40.6	4.76	45.13	9.52	85.73	−0.053	−0.333
* A. formosae*	2163	38.93	4.72	44.85	11.51	83.77	−0.071	−0.418
* P. distinctalis*	2174	41.31	5.34	44.02	9.34	85.33	−0.032	−0.272
* L. haraldusalis*	2121	442.2	4.67	43.33	9.81	85.53	4.664	−0.355
* B. thibetaria*	2241	45.52	4.77	39.58	10.13	85.1	0.070	−0.360
* R. menciana*	2147	43.04	4.84	40.71	11.41	83.74	0.028	−0.404
* B. mori*	2161	43.73	4.58	41.09	10.6	84.82	0.031	−0.397
* S. morio*	2152	41.73	4.83	43.08	10.36	84.8	−0.016	−0.364
* S. lechriaspis*	2160	41.71	4.95	43.84	9.49	85.56	−0.025	−0.314
* C. benjaminii*	2132	41.7	4.88	43.76	9.66	85.46	−0.024	−0.329
AT RICH								
* L. salicis*	325	34.46	2.46	57.23	5.85	91.69	−0.248	−0.408
* C. agnata*	334	46.71	1.5	46.71	5.09	93.41	0.000	−0.545
* H. cunea*	357	45.66	1.12	49.3	3.92	94.96	−0.038	−0.556
* G. menyuanensis*	449	43.65	2.45	49.67	4.23	93.32	−0.065	−0.266
* L. dispar*	371	44.74	2.43	49.6	3.23	94.34	−0.052	−0.141
* E. pseudoconspersa*	388	43.56	2.32	50.26	3.87	93.81	−0.071	−0.250
* G. argentata*	340	43.24	1.47	52.06	3.24	95.29	−0.093	−0.376
* A. formosae*	482	42.95	2.9	49.79	4.36	92.74	−0.074	−0.201
* P. distinctalis*	349	46.13	1.15	49	3.72	95.13	−0.030	−0.528
* L. haraldusalis*	310	45.81	0.97	50.32	2.9	96.13	−0.047	−0.499
* B. thibetaria*	350	44.29	2.57	48.29	4.86	92.57	−0.043	−0.308
* R. menciana*	357	43.7	3.36	47.34	5.6	91.04	−0.040	−0.250
* B. mori*	494	44.74	1.82	50.61	2.83	95.34	−0.062	−0.217
* S. morio*	316	44.3	2.53	48.42	4.75	92.72	−0.044	−0.305
* S. lechriaspis*	441	40.36	2.49	52.38	4.76	92.74	−0.130	−0.313
* C. benjaminii*	293	46.42	3.07	45.73	4.78	92.15	0.007	−0.218

**Table 3:** Details of the lepidopteran mitogenomes used in this study.

Superfamily	Family	Species	Size (bp)	GenBank No.
Bombycoidea	Bombycidae	*Bombyx mori*	15666	KM875545.1
		*Rondotia menciana*	15301	KC881286.1
	Saturniidae	*Actias selene*	15,236	NC_018133
		*Antheraea pernyi*	15,566	AY242996
		*Antheraea yamamai*	15,338	NC_012739
	Sphingidae	*Sphinx morio*	15299	KC470083.1
		*Manduca sexta*	15,516	NC_010266
Noctuoidea	Lymantriidae	*Lymantria dispar*	15507	GU994783.1
		*Gynaephora menyuanensis*	15770	KC185412.1
		*Euproctis pseudoconspersa*	15461	KJ716847.1
		*Leucoma salicis*	15334	This study
	Noctuidae	*Ctenoplusia agnata*	15261	KC414791.1
		*Agrotis ipsilon*	15,377	KF163965
	Nolidae	*Eutelia adulatricoides*	15,360	KJ185131
		*Gabala argentata*	15,337	KJ410747
	Erebidae	*Amata formosae*	15463	KC513737
		*Hyphantria cunea*	15481	GU592049.1
	Notodontidae	*Phalera flavescens*	15,659	NC_016067
		*Ochrogaster lunifer*	15,593	NC_011128
Geometroidea	Geometridae	*Apocheima cinerarium*	15,722	KF836545
		*Biston thibetaria*	15,484	KJ670146.1
Pyraloidea	Crambidae	*Chilo suppressalis*	15,395	NC_015612
		*Diatraea saccharalis*	15,490	NC_013274
		*Paracymoriza distinctalis*	15354	KF859965.1
	Pyralidae	*Lista haraldusalis*	15213	NC_024535
Tortricoidea	Tortricidae	*Spilonota lechriaspis*	15368	HM204705.1
		*Grapholita molesta*	15,717	NC_014806
Papilionoidea	Papilionidae	*Papilio maraho*	16,094	NC_014055
		*Teinopalpus aureus*	15,242	NC_014398
	Nymphalidae	*Apatura ilia*	15,242	NC_016062
		*Apatura metis*	15,236	NC_015537
		*Fabriciana nerippe*	15,140	NC_016419
		*Argynnis hyperbius*	15,156	NC_015988
Hepialoidea	Hepialidae	*Thitarodes renzhiensis*	16,173	NC_018094
		*Ahamus yunnanensis*	15,816	NC_018095
Hesperioidea	Hesperiidae	*Choaspes benjaminii*	15272	JX101620.1

**Table 4:** Details of the primers used to amplify the mitogenome of *L. salicis.*

Primer pair	Primer sequence (5′–3′)	Annealing temperature
F1	TAAAAATAAGCTAAATTTAAGCTT	52 °C
R1	TATTAAAATTGCAAATTTTAAGGA	
F2	AAACTAATAATCTTCAAAATTAT	46 °C
R2	AAAATAATTTGTTCTATTAAAG	
F3	ATTCTATATTTCTTGAAATATTAT	46 °C
R3	CATAAATTATAAATCTTAATCATA	
F4	TGAAAATGATAAGTAATTTATTT	48 °C
R4	AATATTAATGGAATTTAACCACTA	
F5	TAAGCTGCTAACTTAATTTTTAGT	53 °C
R5	CCTGTTTCAGCTTTAGTTCATTC	
F6	CCTAATTGTCTTAAAGTAGATAA	48 °C
R6	TGCTTATTCTTCTGTAGCTCATAT	
F7	TAATGTATAATCTTCGTCTATGTAA	50 °C
R7	ATCAATAATCTCCAAAATTATTAT	
F8	ACTTTAAAAACTTCAAAGAAAAA	53 °C
R8	TCATAATAAATTCCTCGTCCAATAT	
F9	GTAAATTATGGTTGATTAATTCG	53 °C
R9	TGATCTTCAAATTCTAATTATGC	
F10	CCGAAACTAACTCTCTCTCACCT	58 °C
R10	CTTACATGATCTGAGTTCAAACCG	
F11	CGTTCTAATAAAGTTAAATAAGCA	55 °C
R11	AATATGTACATATTGCCCGTCGCT	
F12	TCTAGAAACACTTTCCAGTACCTC	52 °C
R12	AATTTTAAATTATTAGGTGAAATT	
F13	TAATAGGGTATCTAATCCTAGTT	48 °C
R13	ACTTAATTTATCCTATCAGAATAA	
