# Author Correction: Characterization of the Complete Mitochondrial Genome of *Leucoma salicis* (Lepidoptera: Lymantriidae) and Comparison with Other Lepidopteran Insects

**DOI:** 10.1038/s41598-020-63946-0

**Published:** 2020-04-24

**Authors:** Yu-Xuan Sun, Lei Wang, Guo-Qing Wei, Cen Qian, Li-Shang Dai, Yu Sun, Muhammad Nadeem Abbas, Bao-Jian Zhu, Chao-Liang Liu

**Affiliations:** College of Life Sciences, Anhui Agricultural University, 130 Changjiang West Road, Hefei, 230036 Anhui Province P. R. China

Correction to: *Scientific Reports* 10.1038/srep39153, published online 15 December 2016

The Data Availability section was omitted from this Article. It should read:

“Genome assembly data for this study are deposited in GenBank under accession code MT230535.”

