# Supplementary material for: Characterization of the Complete Mitochondrial Genome of Leucoma salicis (Lepidoptera: Lymantriidae) and Comparison with Other Lepidopteran Insects
Source: Sci Rep. 2016 Dec 15;6:39153. doi: 10.1038/srep39153 (PMC5156926; doi:10.1038/srep39153)
Supplement: Supplementary Table S1 [file srep39153-s1.pdf]

Characterization of the Complete Mitochondrial Genome of *Leucoma salicis* (Lepidoptera: Lymantriidae) and Comparison with Other Lepidopteran Insects

Yu-Xuan Sun[1], Lei Wang[1], Guo-Qing Wei[1], Cen Qian[1], Li-Shang Dai[1], Yu Sun[1],

Muhammad Nadeem Abbas[1], Bao-Jian Zhu[1] and Chao-Liang Liu[1]*

Table S1. The identities of entire mitogenomes from other members of Lymantriidae, with the *L. salicis* mitogenome.

Superfamily	Family	Species	Identity	GenBank No.
Noctuoidea	Lymantriidae	*Leucoma salicis*	100%	This study
		Lachana alpherakii	79%	KJ957168.1
		Gynaephora menyuanensis	78%	KC185412.1
		Euproctis pseudoconspersa	78%	KJ716847.1
		Lymantria dispar	77%	GU994783.1
